# Simultaneous and independent electroencephalography and magnetic resonance imaging: A multimodal neuroimaging dataset

**DOI:** 10.1016/j.dib.2023.109661

**Published:** 2023-10-10

**Authors:** Jonathan Gallego-Rudolf, María Corsi-Cabrera, Luis Concha, Josefina Ricardo-Garcell, Erick Pasaye-Alcaraz

**Affiliations:** Instituto de Neurobiología - Universidad Nacional Autónoma de México, campus Juriquilla. Blvd. Juriquilla 3001, Juriquilla, Santiago de Querétaro, Querétaro, México

**Keywords:** Simultaneous EEG-fMRI, Multimodal imaging, Gradient artifact, Ballistocardiographic artifact, EEG data quality, MRI data quality

## Abstract

We introduce an open access, multimodal neuroimaging dataset comprising simultaneously and independently collected Electroencephalography (EEG) and Magnetic Resonance Imaging (MRI) data from twenty healthy, young male individuals (mean age = 26 years; SD = 3.8 years). The dataset adheres to the BIDS standard specification and is structured into two components: 1) EEG data recorded outside the Magnetic Resonance (MR) environment, inside the MR scanner without image collection and during simultaneous functional MRI acquisition (EEG-fMRI) and 2) Functional MRI data acquired with and without simultaneous EEG recording and structural MRI data obtained with and without the participants wearing the EEG cap. EEG data were recorded with an MR-compatible EEG recording system (GES 400 MR, Electrical Geodesics Inc.) using a 32-channel sponge-based EEG cap (Geodesic Sensor Net). Eyes-closed resting-state EEG data were recorded for two minutes in both the outside and inside scanner conditions and for ten minutes during simultaneous EEG-fMRI. Eyes-open resting-state EEG data were recorded for two minutes under each condition. Participants also performed an eyes opening-eyes closure block-design task outside the scanner (two minutes) and during simultaneous EEG-fMRI (four minutes). The EEG data recorded outside the scanner provides a reference signal devoid of MR-related artifacts. The data collected inside the scanner without image acquisition captures the contribution of the ballistocardiographic (BCG) without the gradient artifact, making it suitable for testing and validating BCG artifact correction methods. The EEG-fMRI data is affected by both the gradient and BCG artifacts. Brain images were acquired using a 3T GE MR750-Discovery MR scanner equipped with a 32-channel head coil. Whole-brain functional images were obtained using a GRE-EPI T2* weighted sequence (TR = 2000 ms, TE = 40 ms, 35 interleaved axial slices with 4 mm isometric voxels). Structural images were acquired using an SPGR sequence (TR = 8.1 ms, TE = 3.2 ms, flip angle = 12°, 176 sagittal slices with 1 mm isometric voxels). This stands as one of the largest open access EEG-fMRI datasets available, which allows researchers to: 1) Assess the impact of gradient and BCG artifacts on EEG data, 2) Evaluate the effectiveness of novel artifact removal techniques to minimize artifact contribution and preserve EEG signal integrity, 3) Conduct hardware/setup comparison studies, 4) Evaluate the quality of structural and functional MRI data obtained with this particular EEG system, and 5) Implement and validate multimodal integrative analysis approaches on simultaneous EEG-fMRI data.

Specifications Table

**Table:** 

Subject	Health and medical sciences – Medical imaging.
Specific subject area	Multimodal neuroimaging dataset consisting of simultaneously and independently collected EEG and MRI data.
Data format	Raw data Both EEG and MRI data are organized according to the BIDS standard specification. Structural and functional Magnetic Resonance Images are provided as compressed nifti files (.nii.gz). EEG data files are provided in the EEGLAB structure (.set and .fdt files). The ECG data is included as an additional channel in the EEG dataset. Filtered data For the simultaneous EEG-fMRI acquisitions, we provide EEG data before and after removing the gradient artifact with an average artifact template subtraction approach, implemented in the Net Station software package v. 5.3.
Type of data	1 Tables (2) 2 Fig. (3)
Data collection	EEG data were acquired with a GES 400 MR-compatible recording system (Electrical Geodesics Inc.) including a 32-channel Geodesic Sensor Net, a Net Amps 400 amplifier inside a Field Isolation Containment System for MR use, and a Net Amps clock synchronizer to sync the acquisition of EEG and fMRI data. EEG were recorded using the Net Station software v. 5.3. Electrocardiographic data was collected using MR-compatible patch electrodes. Structural and functional brain MRIs were acquired using a 3T General Electric MR750-Discovery scanner equipped with a 32-channel head coil.
Data source location	Institution: Instituto de Neurobiología, Universidad Nacional Autónoma de México, campus Juriquilla. City/Town/Region: Juriquilla, Santiago de Querétaro, Querétaro. Country: Mexico. Latitude and longitude (and GPS coordinates) for collected samples/data: 20°42′18.8"N 100°26′41.7"W 20.705231, -100.444925
Data accessibility	All the data presented in this manuscript are hosted in a public Mendeley Data repository. Repository name: Simultaneous EEG-fMRI dataset. Data identification number: 10.17632/crhybxpdy6.2. Direct URL to data: https://data.mendeley.com/datasets/crhybxpdy6/2.
Related research article	J. Gallego-Rudolf, M. Corsi-Cabrera, L. Concha, J. Ricardo-Garcell, E. Pasaye-Alcaraz, Preservation of EEG spectral power features during simultaneous EEG-fMRI. Front. Neurosci. 16:951321 (2022). https://doi.org/10.3389/fnins.2022.951321.

## 1 Value of the Data

• Open access multimodal neuroimaging datasets are scarce. This is one of the largest simultaneous EEG-fMRI datasets available, also providing independently collected EEG and functional/structural MRI data. Considering the challenges of simultaneous EEG-fMRI (i.e., data artifacts, low reproducibility), this dataset represents a valuable tool for the neuroimaging community seeking to test innovative data preprocessing and multimodal integration methodologies.
• This dataset benefits the existing EEG-fMRI scientific community by providing data that can be used for implementing novel analytical approaches and validate previous findings from the EEG-fMRI literature. Considering the relatively limited accessibility to simultaneous EEG-fMRI recording setups, we hope this dataset will also incentivize other scientists to work with multimodal EEG-fMRI data, contributing to the growing collective effort to overcome the challenges associated with this technique.
• This dataset may be used to further characterize the properties of the gradient and BCG artifacts and their impact on EEG signal properties, to compare the artifacts’ profile across hardware setups, to assess the impact of the EEG hardware on MRI data quality, to test novel EEG/MRI artifact correction approaches, and to perform multimodal analyses that integrate the information provided by electrophysiological and hemodynamic signals during resting-state.

## 2 Data Description

**Fig. 1:**
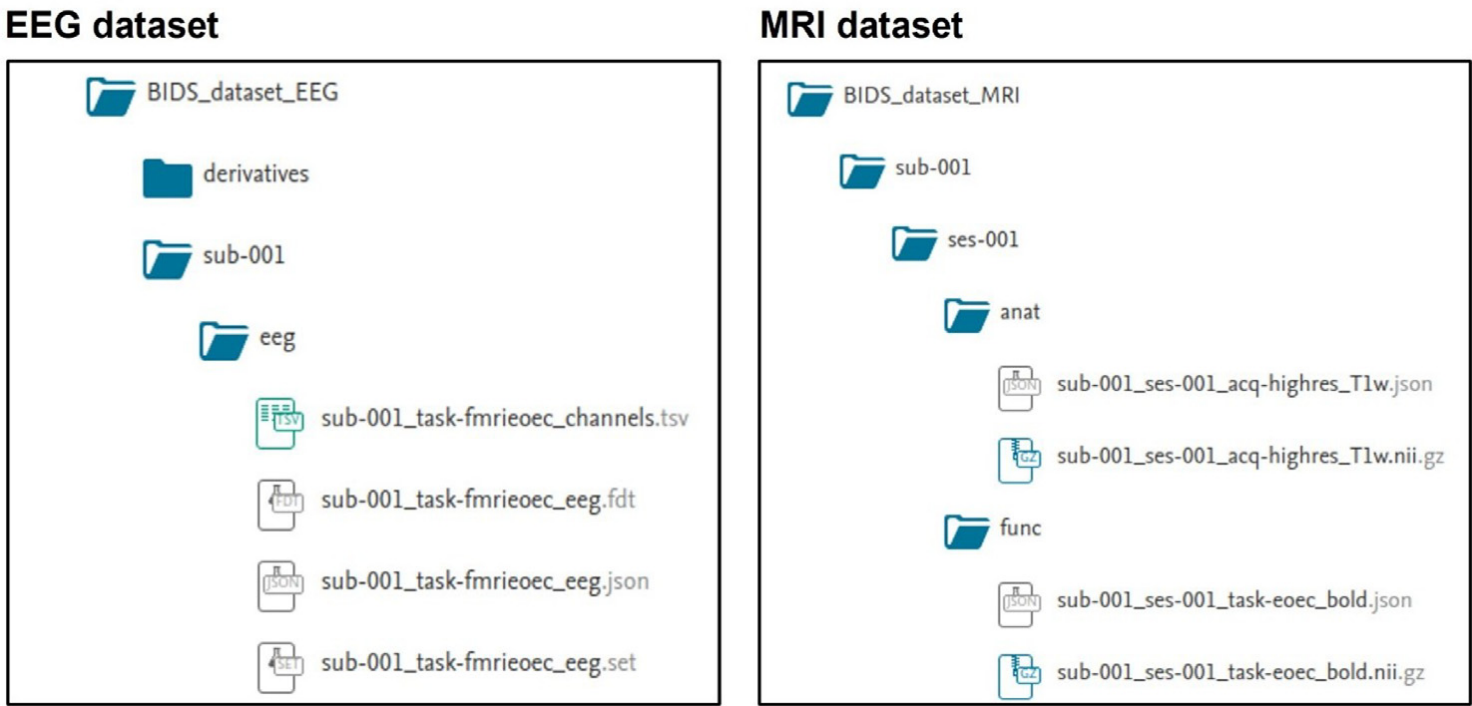
File organization within the dataset. Schematic representation of the data repository structure. The dataset is integrated by the EEG (left panel) and the anatomical/functional MRI data (right panel), collected independently and simultaneously. The data are organized according to the BIDS standard specification and hosted in a public data repository (https://data.mendeley.com/datasets/crhybxpdy6/2).

The data presented here supports our related research article “Preservation of EEG spectral power features during simultaneous EEG-fMRI” [1] and is hosted in a Mendeley Data open access repository [2]. The dataset follows the BIDS standard specification [3,4] and is separated into two components, one for the EEG and one for the MRI data. Fig. 1 shows the organization of the EEG and MRI data files within the dataset.

### 2.1 EEG dataset

The root BIDS_dataset_EEG directory (Fig. 1, left panel) contains the folders with the EEG data of each participant (labelled, sub-0XX), a README and a dataset description file providing an overview of the dataset content, a participant's information file containing the ids, age, and sex of the participants and the required bidsignore file. The folder of each participant comprises their EEG data (eeg folder) and the accompanying json and tsv files describing the experimental conditions for each EEG file. The EEG data recorded outside the MR environment consists of two minutes of eyes-closed (EC) resting-state, followed by two minutes of eyes-open (EO) resting-state and two minutes of an eyes opening-eyes closure (EO-EC) task, where participants alternated between EO and EC states in twenty-second intervals. These data were concatenated into a single file, labelled *task-outside_eeg*. The EEG data obtained inside the MR scanner without image acquisition includes two minutes of EC and two minutes of EO resting-state data. These data were also concatenated into a single file, labelled *task-inside_eeg*. For the EEG data acquired simultaneously with fMRI, recordings were saved in three separate files: ten minutes of EC resting-state (labelled *task-fmrirestingec*), two minutes of EO resting-state (labelled *task-fmrirestingeo)* and four minutes of the same EO-EC task performed outside the scanner (labelled *task-fmrieoec*). Each of the recordings consists of a pair of set and fdt files (eeglab data structure) containing the EEG data, a json file including data acquisition parameters and a tsv file containing the list of EEG channels included in the recordings. For the simultaneous EEG-fMRI acquisitions we also provide the gradient artifact-corrected EEG set and fdt files, which are stored in the derivatives folder under the root BIDS_dataset_EEG directory. These files can be identified with the same labels plus the _gac (gradient artifact corrected) suffix. A summary of the EEG data files included in the dataset is presented in Table 1.

**Table 1:** **EEG sessions included in the dataset.** EEG was collected in three different settings: outside the MR scanner, inside the MR scanner without acquiring images and during simultaneous EEG-fMRI. The table shows the duration (in minutes) of the EC resting-state, EO resting-state and EO-EC task conditions. The last column shows the naming convention for the files included in the EEG BIDS dataset. EEG data from the outside and the inside scanner conditions were saved as single six- and four-minutes files, respectively. EEG recordings acquired simultaneously with fMRI data were stored as separate files.

	Eyes-closed resting-state	Eyes-open resting-state	EOEC-task	Filenames
Outside EEG	2 min	2 min	2 min	Single file: *task-outside_eeg*
Inside EEG	2 min	2 min	–	Single file: *task-inside_eeg*
EEG-fMRI	10 min	2 min	4 min	Separate files: *fmrirestingec, fmrirestingeo, fmrieoec*

**Fig. 2:**
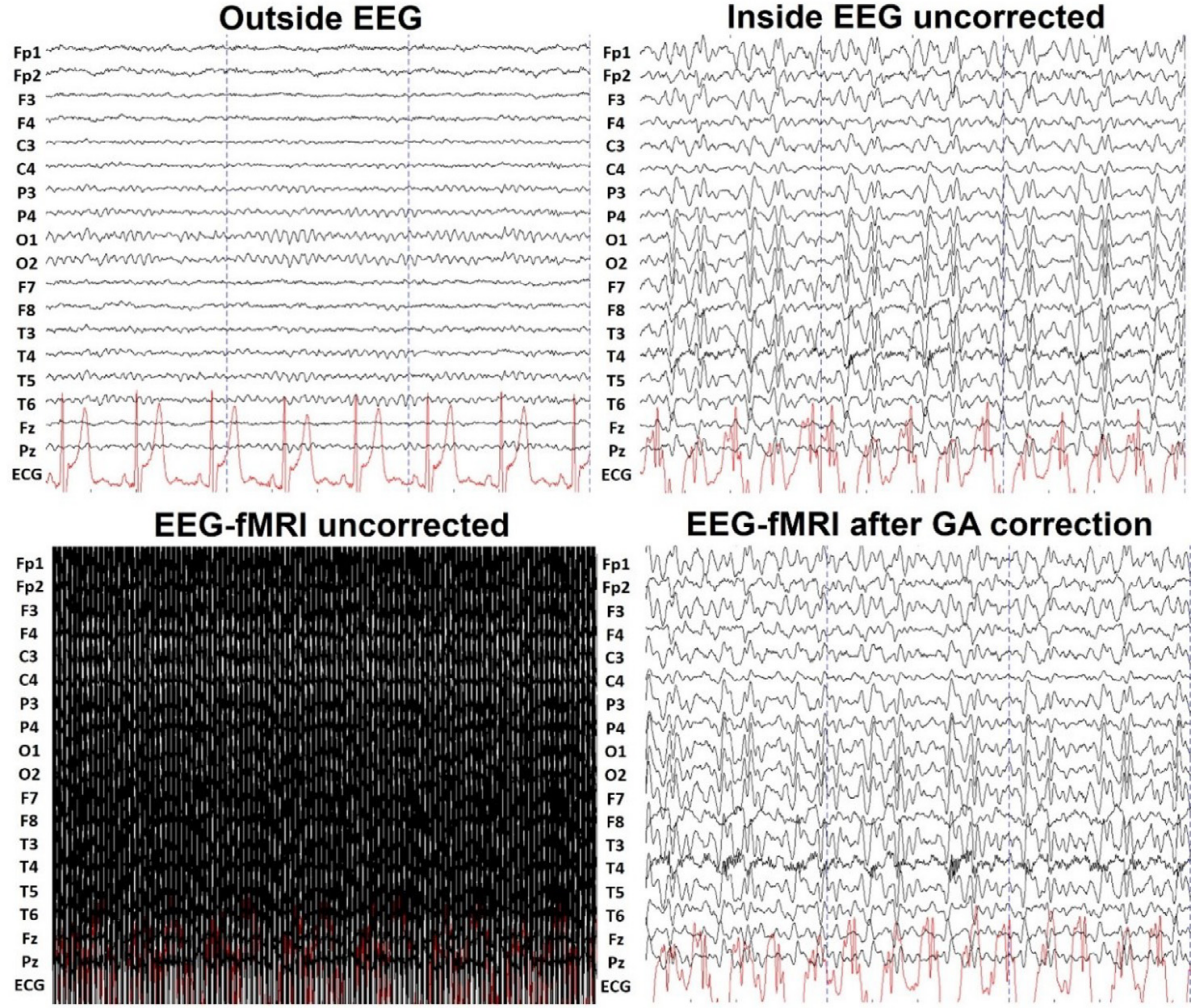
Representative example of the EEG data. Six-seconds of EEG data recorded from a representative participant (sub-008) under each condition. The top row shows the EEG traces recorded outside (left) and inside (right) the MR scanner. The bottom row shows the EEG data recorded during simultaneous EEG-fMRI, before (left) and after (right) removing the gradient artifact correction. The last channel corresponds to the ECG data.

Fig. 2 shows an example of six-seconds of eyes-closed EEG signal from a representative individual (sub-008), recorded in each of the three conditions: outside the scanner, inside the scanner without image acquisition and during simultaneous fMRI acquisition. The outside scanner recordings provide an EEG signal free of MR-related artifacts (top-left). The inside scanner condition records the contribution of the ballistocardiographic (BCG) artifact without the presence of the gradient artifact (GA), as no MRI is acquired (top-right). The data from the EEG-fMRI condition is contaminated by both the GA and the BCG artifact (bottom-left). The bottom-right panel shows an example of the EEG data after removing the GA artifact with average artifact subtraction. The last channel corresponds to the ECG signal time series.

### 2.2 MRI dataset

Similar to the EEG dataset, the root BIDS_dataset_MRI directory (Fig. 1, right panel) contains the folders with the MRI data for each participant, a README and dataset description files, and the tsv and json participant files. The additional json files labelled *task-rest_bold* and *task-eoec_bold* contain some of the imaging parameters of the EC resting-state and EO-EC task conditions, respectively. Each participant's directory contains two subfolders: Session 1 includes the MRI data acquired concurrently with EEG and session 2 the images collected without the EEG cap. Each session contains anatomical and functional MRI data folders and a pair of tsv and json files with the information of the scanning session. The functional data from session 1 includes a pair of compressed nii and json files for the EC resting-state (*task-rest_bold*) and the EO-EC task (*task-eoec_bold*) data. The EO-EC task includes an additional tsv file (*task-eoec_events*), containing the time onset for the EO and EC alternations. The anatomical data folder contains the structural MRI scan acquired while the participants were wearing the EEG cap. Session 2 includes the functional data collected during EC resting-state without simultaneous EEG and the structural scan acquired without the EEG cap. No EO-EC fMRI data were acquired without simultaneous EEG recording. The only missing session in the dataset is the EC resting-state fMRI data from session 1 of sub-019, as there was a problem with the MRI sequence and the file was not properly reconstructed after data acquisition. A detailed description of the parameters for the functional and structural MRI sequences is presented in Table 2.

**Table 2:** **MRI sessions included in the dataset.** For the first part of the protocol (ses-001) MRIs were collected simultaneously with EEG. Functional MRI acquisitions (EC resting-state, EO resting-state and EOEC-task) were collected before the structural MRI. For the second part of the experiment (ses-002) the same sequences were used to obtain the EC resting-state fMRI and the structural image without the EEG cap. The file naming convention and the sequence parameters are presented for the functional and structural acquisitions, respectively.

	Functional MRI			Structural MRI
	Eyes-closed resting-state	Eyes-open resting-state	EOEC-task	Eyes-closed
With EEG (ses-001)	10 min	2 min (not included)	4 min	5 min 30 s
Without EEG (ses-002)	10 min	—	—	5 min 30 s
Filenames	task-rest_bold.nii	—	task-eoec_bold.nii	acq-highres_T1w.nii
Sequence parameters	Type=GRE-EPI T2*, FoV=25.6cm, Matrix=64 × 64, TR=2000ms, TE=40ms, 35 slices (axial, interleaved, bottom-up), Voxel size=4 mm³			Type=SPGR T1, FoV=25.6cm, Matrix=256 × 256, TR=8.1ms, TE=3.2ms, 176 sagittal slices, Voxel size = 4 mm³

**Fig. 3:**
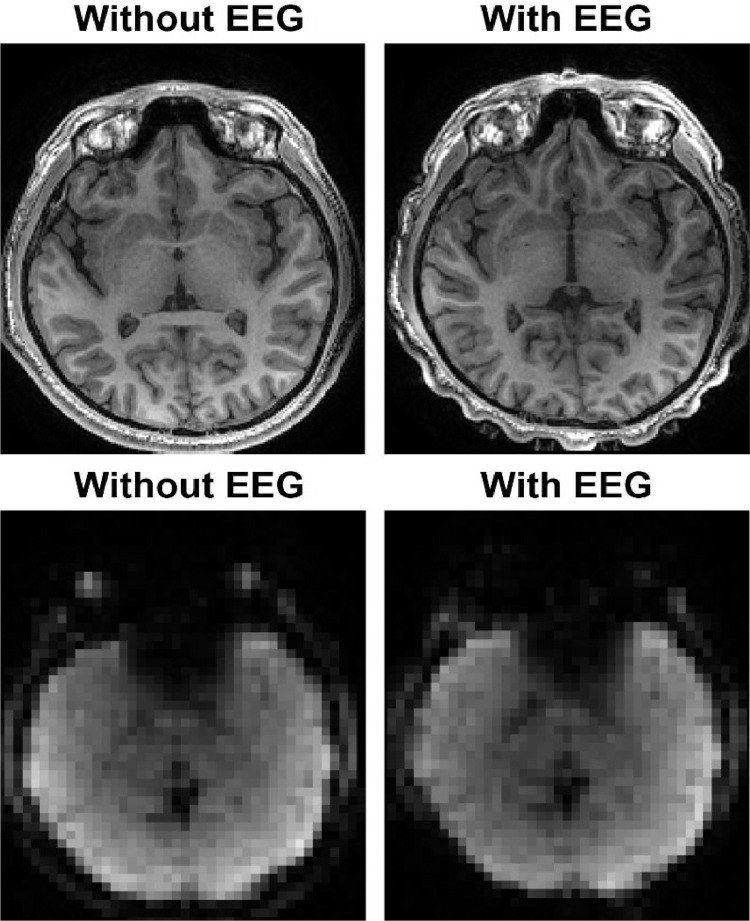
Representative example of the MRI data. MRI data from a representative individual (sub-001). The top row shows an axial slice of the structural MRI data acquired with and without the EEG cap. The bottom row shows an axial slice of a single time point of the fMRI data, acquired with and without simultaneous EEG recording.

Fig. 3 shows an example of the structural (top) and functional (bottom) MRIs from a representative individual (sub-001). The images on the left show the data acquired without the EEG cap and the images on the right the data collected with the EEG cap. The presence of the EEG electrodes can be clearly appreciated in the structural scan acquired with the EEG cap (top-right).

## 3 Experimental Design, Materials and Methods

The primary objective behind collecting this dataset was to gather simultaneously and independently acquired EEG and MRI data from the same individuals. This approach enables to assess the quality of EEG and MRI data obtained simultaneously [5], to compare various methodological approaches for artifact removal in each data modality [6] and to conduct multimodal integrative analyses of electrophysiological and hemodynamic signals [7,8]. Our sample included 20 healthy, young male graduate students from the Universidad Nacional Autónoma de México (UNAM), campus Juriquilla in Queretaro, Mexico (mean age = 26 years; SD = 3.8 years). Potential participants were administered the Spanish version of the MINI International Neuropsychiatric Interview [9], to ensure the absence of any neurological/psychiatric diseases or a substance abuse history. Participants also completed a brief checklist to rule out any contraindications for undergoing MRI scans. Participants meeting these criteria were invited to join the study and were required to provide informed consent, agreeing to their de-identified data to be used in the primary research study and to be shared in the open repository.

On the day of the experiment, participants were instructed to arrive around 5:30 pm with clean hair and wearing comfortable, non-metallic clothing. They were also required to avoid alcohol, coffee and sleep deprivation 12 hours prior to the experiment. EEG data were recorded using a GES 400 MR system (Electrical Geodesics Inc. [EGI], Eugene, OR, USA) equipped with a 32-channel MR-compatible Geodesic Sensor Net EEG cap, a Net Amps 400 amplifier placed inside a Field Isolation Containment System for MR use, and a Net Amps clock synchronizer device to sync the acquisition of EEG and fMRI data (an essential step for effective gradient artifact removal [5,6]). Individual head measurements were obtained to select the appropriate EEG cap size for each participant. The sponge-based EEG cap was immersed in a K^+^Cl^−^ solution to improve electrode conductivity, and it was placed over the participants’ head, following the instructions provided in the EGI manuals. After adjusting each electrode individually to ensure accurate placement and impedance values below 50 k-ohms, a silk mesh was positioned over the cap to prevent electrode displacement and improve EEG data quality [6,10]. EEG signals were recorded with a sampling rate of 1000 Hz, and Cz was used as the reference electrode. To assist BCG artifact removal, electrocardiographic (ECG) signals were recorded using MR-compatible patch electrodes placed over the heart (active) and the medial end of the left collarbone (reference). Both EEG and ECG data were recorded using Net Station software v. 5.3, also distributed by EGI.

We first recorded EEG data outside the MR environment, with participants lying down in the same position as inside the MR scanner. We collected two minutes of eyes-closed (EC) and two minutes of eyes-open (EO) resting-state EEG, followed by a two-minute block-design task where participants alternated between EC and EO every twenty seconds, as verbally indicated by the experimenter. Following the outside scanner EEG acquisitions, the participants were taken into the MR scanner. The EEG amplifier (inside the Field Isolation Containing System) was placed on a non-metallic support table adjacent to the scanner bore, behind the 400 Gauss isointensity line, and plugged to the recording computer and the sync clock device via optic fiber cables. To ensure consistent positioning across individuals, the isocenter of the magnet was aligned with the nasion of each participant. EEG leads were aligned parallel to the B0 magnetic field and meticulously checked for loops to minimize EEG artifacts and radiofrequency-induced heating [5,6]. Sandbags and tape were employed to limit movement of the EEG leads, while soft pads were used to reduce head movement within the head coil [10]. Throughout the session, scanner lights and ventilation systems were kept off to prevent further artifacts on the EEG signal [10,11]. The magnet's cold head Helium pump remained operational in compliance with facility protocols, which might induce additional artifacts in the EEG signal [6,12]. Once participants were comfortable inside the MR scanner, we recorded two minutes of EC and two minutes of EO resting-state EEG without image acquisition. This inside scanner condition captures the contribution of the BCG but not the gradient artifact, as the former depends on the static B0 magnetic field, and the latter is induced by rapid magnetic field gradients switching during image acquisition [5,6].

After collecting the inside scanner EEG data without image acquisition, we initiated the simultaneous EEG-fMRI protocol. Brain images were obtained with a Discovery-MR750 3.0 T MR scanner (General Electric, WI, USA), equipped with a 32-channel-array head coil. Blood-oxygen level-dependent (BOLD) contrast functional images were obtained using a Gradient Recalled Echoplanar Imaging (GRE-EPI) T2*-weighted sequence, with a spatial resolution = 4 × 4 × 4 mm³ voxels, TR = 2000 ms, TE = 40 ms, 35 axial slices obtained using bottom-up interleaved acquisition, and a flip angle = 90°. Anatomical images were acquired using a Spoiled Gradient Recalled (SPGR) sequence, with a spatial resolution of 1 × 1 × 1 mm³ voxels, TR = 8.1 ms, TE = 3.2 ms, 176 sagittal slices, and flip angle = 12°. We recorded ten minutes of EC resting-state, followed by a brief two-minute EO resting-state acquisition. Due to its brief duration, the fMRI data from the EO resting-state acquisition is not included in the dataset; only the EEG data is provided. Subsequently, participants were instructed to perform the same EO-EC task described earlier for a total of four minutes, comprising six EC and six EO blocks. After the fMRI acquisition, we obtained the structural MRI scan while the participants were still wearing the EEG cap. Participants were then taken out of the MR scanner room to have the EEG cap and ECG electrodes removed. The EEG amplifier, supporting table and optic fiber cables were removed from the MR scanner room, before bringing the participant back into the scanner to repeat the ten-minute EC resting-state and anatomical scan acquisitions without the EEG cap. After concluding the MRI acquisition without EEG, participants were escorted out of the MR-room, marking the end of the experiment. The entire session lasted approximately two and a half hours.

### 3.1 Preparation of the open dataset

The raw EEG files were exported from their native format (mff) to the EEGLAB [13] data structure (set and fdt files) using the corresponding function in the Net Station software. We then imported the data into EEGLAB and specified the channel locations and labels according to the corresponding 32-channel EGI montage (included as part of the default eeglab chanlocs files) and indicated that Cz was used as the reference electrode. BIDS sidecar files were generated using the data2bids function included in the Fieldtrip software package [14]. For the derivative gradient artifact corrected EEG files, the gradient artifact was removed using the Net Station software v. 5.3, prior to importing the data into EEGLAB. We used an average artifact subtraction approach with a sliding window to generate a moving template of the artifact across five fMRI volumes, which we then subtracted from the EEG signal recorded on each channel. This method relies on an accurate synchronization between the EEG and fMRI acquisition, which was achieved by using the TR pulses generated by the MR scanner to create event labels and align the occurrence of the artifact to generate the template.

For the MRI data, raw DICOM files were converted to the compressed nifti file format and organized according to the BIDS standard using the heuristic DICOM converter [15]. In accordance with data sharing principles and to ensure that the anatomical MRI data cannot be used for identification purposes, we used the *mri_deface* tool from the FreeSurfer software distribution [16] to remove the facial features of the participants from each slice of the anatomical MRI volumes.

## 4 Limitations

Although our dataset represents one of the largest open access simultaneous EEG-fMRI data repositories available, we acknowledge that the sample size (n=20) is still relatively small for statistical purposes. Additionally, given that the aim of the main research study was to evaluate the preservation of EEG spectral features after artifact removal, we opted for recruiting only male participants to avoid additional variability in resting-state electrophysiological spectral features related to hormonal fluctuations during the menstrual cycle in women [17]. The lack of inclusion of female participants represents an important limitation of this dataset. Even though we put a lot of effort into ensuring consistency across participants, subtle differences in head shape, positioning and movement across the EEG-fMRI scanning session may result in higher variability of the BCG artifact profile across individuals. Finally, it is important to note that the generalizability of the results derived from multimodal analysis using this dataset may be limited due to hardware/setup differences with respect to other studies.

## Ethics Statement

Before being enrolled into the study, the research protocol was explained to the participants both verbally and through an informed consent form. Only participants who gave their consent to be part of the experimental protocol and allowed their de-identified data to be used for main project analysis and shared in an open repository were recruited into the study. This research project was conducted in accordance with the principles of the Declaration of Helsinki for experiments involving human participants and was approved by the Bioethics Committee of the Instituto de Neurobiología, Universidad Nacional Autónoma de México, Campus Juriquilla (Protocol number: 057HRM).

## CRediT authorship contribution statement

**Jonathan Gallego-Rudolf:** Conceptualization, Methodology, Software, Validation, Formal analysis, Investigation, Resources, Data curation, Writing – original draft, Writing – review & editing, Visualization, Project administration. **María Corsi-Cabrera:** Conceptualization, Methodology, Validation, Writing – review & editing, Supervision. **Luis Concha:** Conceptualization, Methodology, Validation, Investigation, Resources, Writing – review & editing, Funding acquisition. **Josefina Ricardo-Garcell:** Conceptualization, Methodology, Validation. **Erick Pasaye-Alcaraz:** Conceptualization, Methodology, Validation, Investigation, Resources, Data curation, Supervision, Project administration, Funding acquisition.

## Data Availability

Simultaneous EEG-fMRI dataset (Original data) (Mendeley Data).
